# Sharing and reuse of mental health research data: Introducing the HeSANDA mental health node

**DOI:** 10.1177/10398562251382462

**Published:** 2025-09-27

**Authors:** Alison R Yung, Nemanja Zivanov, Marko Milicevic, Kristan Kang, Andrew Thompson, Alyna Turner, Ayla Barutchu, Lourdes Llorente, Delyth Samuel, Olivia M Dean, Rhys Williams, Suzie Lavoie

**Affiliations:** Institute of Mental and Physical Health and Clinical Translation (IMPACT), 2104 Deakin University, Geelong, VIC, Australia; School of Health Sciences, University of Manchester, Manchester, UK; 3487 Barwon Health, Geelong, VIC, Australia; Institute of Mental and Physical Health and Clinical Translation (IMPACT), 2104 Deakin University, Geelong, VIC, Australia; 607097 Australian Research Data Commons, Australia; Orygen, Parkville, VIC, Australia; Centre for Youth Mental Health, The University of Melbourne, Parkville, VIC, Australia; Institute of Mental and Physical Health and Clinical Translation (IMPACT), 2104 Deakin University, Geelong, VIC, Australia; Institute of Mental and Physical Health and Clinical Translation (IMPACT), 2104 Deakin University, Geelong, VIC, Australia; 3487 Barwon Health, Geelong, VIC, Australia; Deakin Research Innovations, Burwood, VIC, Australia; Institute of Mental and Physical Health and Clinical Translation (IMPACT), 2104 Deakin University, Geelong, VIC, Australia; 56369 Florey Institute of Neuroscience and Mental Health, Parkville, VIC, Australia; 607097 Australian Research Data Commons, Australia; Orygen, Parkville, VIC, Australia; Centre for Youth Mental Health, The University of Melbourne, Parkville, VIC, Australia

**Keywords:** data sharing, data reuse, trusted research environment, secure research environment

## Abstract

**Introduction:**

Data sharing is the practice of making de-identified participant-level data available for use by other researchers. It increases the potential of a dataset to answer new questions, accelerates knowledge creation and increases research integrity by allowing conclusions to be replicated, verified or corrected. Data sharing helps fulfil the ethical obligation to make the most of research participants’ contributions to science.

**Analysis and Evidence:**

There is evidence that research participants and the general public are supportive of data sharing. However, those who conducted the original studies may be reluctant to share data, and datasets may be difficult to access, and there may be ethical and governance concerns.

**Discussion:**

This paper describes the Mental Health Node, an Australian Government initiative that aims to increase mental health data sharing. The Mental Health Node works with primary researchers (those who conduct original studies), and secondary researchers (those who reuse data generated by others) to promote ethical data sharing that respects the role of primary researchers and the privacy concerns of research participants.

**Conclusion:**

Primary and secondary researchers can collaborate to maximise the value of data collected. This paper includes recommendations for good practice in data sharing and links to resources.

## Introduction

Data sharing is the practice of making de-identified participant-level data available for use by other researchers. The benefits of sharing data from both clinical trials and cohort studies are well-documented^1–8^ and are summarised in Box 1. Mental health clinical trials play a key role in influencing evidence-based practice and policy but are often expensive, difficult to run and frequently have small sample sizes.^9^ Hence, the benefit of reusing and combining datasets is clear. As for cohort studies, combining datasets provides advantages when investigating rare exposures or outcomes. It also enhances confidence in the validity of findings, given that populations, sample ascertainment and loss to follow-up data are likely to vary across cohorts, even in those studies that aim to study the same exposures and outcomes.^10^

**Box 1.** Benefits of sharing clinical trials data.

Knowledge creation
• Enables individual trials to be combined to create a larger sample size enabling detection of small effects. This may result in stronger evidence about the efficacy (or otherwise) of an intervention.
• Enables existing research findings to be extended
• Enables testing of new hypotheses that are different from those of the original study or studies
• Accelerates research discovery
• May enable virtual trials^25^ to be conducted, which use mathematical modelling to simulate how participants with different characteristics might respond to an intervention
Improving integrity of research
• Allows conclusions to be re-examined, replicated, verified or corrected
• Ensures that data are correctly interpreted, and improves research integrity
• Increases transparency in research
Ethical issues
• Deters inaccurate reporting of trial results
• Helps fulfil the ethical obligation to make the most of research participants’ contributions to science
• Reduces the burden on research participants
Logistic issues
• Unnecessary repetition of studies can be avoided
• Reduces research waste by using data already collected
• Fosters collaboration among researchers
• Provides value for money
• May result in more papers and citations for those who share datasets
• It is cheaper and quicker than conducting a primary data collection study

Indeed, there is growing recognition that data produced by publicly funded research should be made available for reuse. For example, it is now an expectation of the United States National Institutes of Health (NIH) that all of their grantees share research data.^11^ In Australia, the National Health and Medical Research Council (NHMRC) Open Access Policy 2022 (updated November 2023) strongly encourages researchers to take reasonable steps to share research data arising from NHMRC-funded research.^12^

This paper describes the Health Studies Australian National Data (HeSANDA) Mental Health Node, an Australian Government initiative that aims to increase data sharing by promoting the benefits of data sharing for primary and secondary researchers and research participants while addressing potential issues associated with this practice.

## Risk and concerns about data sharing

Despite the benefits of secondary use of data, there are associated risks and concerns, particularly in the current climate of data security and data breaches. Research conducted into attitudes about data sharing has found a number of concerns expressed by research participants,^9^ the general public,^4,5^ and the researchers who conducted the primary studies (‘primary researchers’).^13^ These are summarised in Box 2. Potential secondary data users also face barriers in finding, accessing and analysing datasets.^3^ These are shown in Box 3.

**Box 2.** Concerns about data sharing.

Clinical trials participants’ and general public concerns
• Concerns that privacy cannot be adequately protected
• Exploitation of data for profit
• Theft of data
• Data will be used for marketing or insurance purposes
Primary researchers’ concerns about data sharing
The above concerns, as well as:
• Time consuming preparation of data for sharing
• Shared data not being published by collaborators
• Misinterpretation of data by secondary researchers who may not fully understand them
• Concerns about the expertise and motivations of data requestors
• Lack of acknowledgement of the data creators
• Theft of ideas
• Rival researchers getting easy publications
• People being deterred from participating in clinical trials due to concerns about their data being shared

**Box 3.** Barriers to secondary data use.

Difficulty finding datasets
• Knowing where to look • Ambiguous terminology and data dictionaries • Data stored in customised databases • Difficulty accessing the original research protocol
Difficulty requesting access to datasets
• No clear point of contact • Point of contact has moved on • Point of contact does not reply to requests • Reluctance of primary researchers to share data • Unclear process for requesting data • May be costly
Difficulty gaining ethical and governance approvals • Time consuming and burdensome • Ethics committees reluctant to approve waivers of consent • Ambiguity within guidelines from Government, NHMRC or Ethics Committees regarding sharing anonymised data and governance/ethics processes
Difficulty analysing data
• May require use of a trusted research environment which may be expensive to build or access • Variables of interest may not have been measured
Difficulty interpreting data
• Shared data is not generalisable to the secondary user’s local context • Lack of knowledge about the cohort, methodology, and dataset (e.g. lack of data dictionaries, poorly kept datasets) • Inconsistencies in quality/cleanliness of datasets

## The HeSANDA Mental Health Node

A new initiative in Australia aims to address the concerns and minimise the barriers noted in Boxes 2 and 3. The Australian Research Data Commons (ARDC) has developed the HeSANDA program to build national infrastructure to support the sharing and reuse of health research data. Nine nodes are funded as part of HeSANDA, of which the Mental Health node is one. See Box 4 for details of all the nodes.

**Box 4.** HeSANDA nodes

Melbourne Academic Centre for Health (MACH) clinical Trials Consortium Node
Mental Health Node
Monash and partners Node
National Cancer Co-operative Trials Group Node
Northern Australian Node
Queensland Node
South Australia Node
Sydney Health Partners Node
Western Australia Node

The Mental Health node is a partnership between the Deakin University, Barwon Health, the Mental Health Australia General Clinical Trials Network (MAGNET), Orygen and the Australian Early Psychosis Collaborative Consortium (AEPCC).^14^ Together with the ARDC, the Mental Health Node aims to address the concerns of primary researchers, the barriers faced by potential secondary data users and the concerns of trial participants and the general public.

### Addressing the concerns of primary researchers

The Mental Health Node team has emphasised to primary researchers that they can specify the conditions under which they are prepared to share data. This could include that they are involved in the data analysis, interpretation and/or write-up. This is appropriate since, to reuse data collected by others, secondary researchers need contextual information from primary researchers such as documentation about protocols, sample ascertainment, and the procedures used for collecting and processing the data.^15^ Therefore, there is a case for potential reusers to collaborate with primary researchers in a ‘win-win’ situation that recognises the substantial role of the primary researchers.^16^

Primary researchers and data custodians also need to be reassured that their datasets can be shared securely, without the possibility that secondary researchers will copy or share the data without permission, or re-identify participants. These concerns can be mitigated by use of ‘trusted research environments’ (TREs). TREs are highly secure controlled computing environments that allow approved researchers to gain access to and analyse data via virtual data analysis workspaces with built-in data analysis tools. TREs ensure that data cannot be copied or exported, and they protect participant privacy. Researchers cannot remove individual participant-level data from a TRE but can export data analysis results after approval from data custodians. The Mental Health Node uses the Secure Health data and Biosamples platform (SHeBa) TRE.^17^

### Addressing the barriers faced by potential secondary data users

Primary researchers and data custodians have been working with the Mental Health Node to make their mental health clinical trial meta-data (information about the data set) findable and searchable on the Health Data Australia^18^ and Mental Health Node.^19^ These websites include an online catalogue with descriptions of Australian mental health clinical trials data, including information about the study, such as the investigators, research questions, study design, sample size, variables measured, as well as the study protocol and other relevant study documents. Importantly, the data access policy of the host institution for the dataset is available, providing guidance for potential secondary users about data access, including ethical and governance requirements. Potential secondary data users can therefore feel confident that the datasets on these websites are potentially available for reuse.

### Addressing the concerns of trial participants and the general public

Despite the issues summarised in Box 2, study participants and the general public are generally supportive of data sharing and reuse.^1^ Research conducted in England found strong support by the general public for data sharing.^20,21^ The majority of survey responders had confidence that their data would be held securely and were supportive of their reuse provided the motivation and methods for this were clear and transparent.^21^ Important exceptions to this positive approach were concerns about data use by commercial interests, particularly by insurance companies, the pharmaceutical industry and marketing companies.^21–23^

In Australia, prior to launching the HeSANDA initiative, the ARDC conducted workshops with the general public and clinical trial participants. In these workshops, participants emphasised the importance of informed consent for sharing data from clinical trials and the need to ensure protection from individual identification throughout the data sharing process. They were overwhelmingly positive about the intent of HeSANDA to facilitate secondary use of data from clinical trials research and the importance of making use of data generated through research.^24^

## Summary and recommendations for researchers

### Recommendations for primary researchers

The decision to share data should be made while the study is being set up. The Participant Information Sheet and Consent Form (PICF) can then include provision for participants to consent (or not) to having their non-identifying data made available for future research studies. The associated Data Management Plan should contain the details on what data will be available for secondary use, data dictionaries, agreements to share study documents including protocols to help secondary users understand the data, and outline governance processes and how the data will be shared. Tips and templates for preparing this documentation are available on the Mental Health Node website under the ‘Resources’ tab. Individual participant data made available for sharing should be de-identified. Data should be shared via a TRE such as SHeBa.

Primary and secondary researchers should enter into data sharing agreements to ensure secondary users are accessing, analysing and publishing results that adhere to institutional and primary researcher policies and any specific requirements.

### Recommendations for secondary researchers

Secondary researchers should collaborate with primary researchers to ensure that they understand the context of the original study, they understand the dataset, and that they give appropriate acknowledgement of the original data creators. They should follow institutional guidelines and policies for data sharing if they exist. Secondary researchers should obtain ethics approval for the use of the secondary data to answer new research questions, and this approval should be provided to the primary researchers. If they are not using a TRE, they should ensure that the data are kept safe at all times, and that they will not try to identify the participants. A guide for secondary users is available from HeSANDA.^3^

### Recommendations for data sharing agreements

A data sharing agreement is an agreement between two or more entities (e.g. universities) that are providing and receiving data. It specifies the conditions under which the data will be shared. We recommend that such an agreement includes names of the secondary research team, which primary researchers should be named as authors on any outputs, and the limitations of the secondary data use. For example, the data sharing agreement may specify how long the data will be available for, whether it is only to be used for non-commercial purposes, any reporting requirements (e.g. updates from the secondary users to the primary researchers) and may include security-related restrictions such as specifications on data transfer, storage, and analysis environments. For example, primary researchers may state that their data set can only be accessed via a specific TRE. Primary researchers should also make clear any costs involved in sharing their data, such as for use of a TRE, and any costs for their time in preparing a data set for reuse. Such a set of rules ensures that all parties are clear about what can and cannot be done with the shared dataset. Guides and templates for creating a data sharing agreement are available on the ARDC and Mental Health Node websites.^2,19^

## Conclusion

The Mental Health Node aims to increase the responsible sharing and reuse of mental health research data in order to accelerate new discoveries and translate research into practice and policy. Through the HeSANDA initiative, we aim to assist and encourage researchers to share and reuse data securely. We also aim to raise awareness about how to make datasets discoverable and searchable and how to find, request and access datasets. Interested readers are invited to access Health Data Australia and the Mental Health Node websites.^18,19^
